# 4,4′-(Phenyl­imino)dibenzaldehyde

**DOI:** 10.1107/S1600536809031924

**Published:** 2009-08-19

**Authors:** Yong-Gang Wang, Xue-Jie Tan, Dian-Xiang Xing

**Affiliations:** a Department of Chemical Industry, Shandong Institute of Light Industry, Jinan 250353, People’s Republic of China

## Abstract

The asymmetric unit of the title compound, C_20_H_15_NO_2_, contains one half-molecule with the central N atom and two C atoms of the benzene moiety lying on a twofold rotation axis. Weak C—H⋯O inter­actions join the mol­ecules together into an infinite three-dimensional network.

## Related literature

The title compound was obtained unintentionally as the product of an attempted purification of tris­(4-formyl­phen­yl)amine, which is used as a building block in materials chemistry (Thomas *et al.*, 2005 ▶). For hydrogen bonding, see: Krishnamohan Sharma & Desiraju (1994 ▶). =
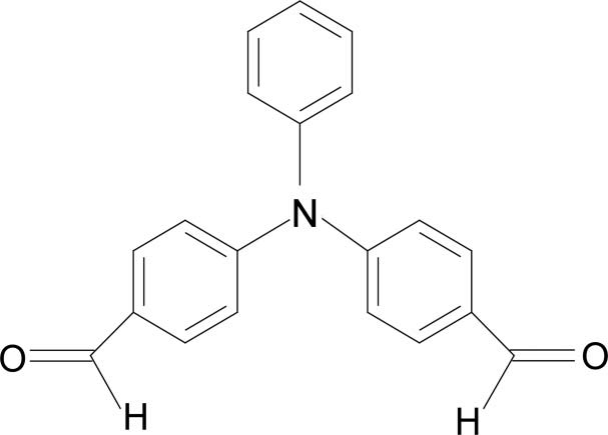


## Experimental

### 

#### Crystal data

C_20_H_15_NO_2_
*M* *_r_* = 301.33
Orthorhombic, 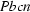

*a* = 8.836 (2) Å
*b* = 9.710 (2) Å
*c* = 18.621 (4) Å
*V* = 1597.6 (6) Å^3^
*Z* = 4
Mo *K*α radiation
μ = 0.08 mm^−1^
*T* = 298 K
0.32 × 0.18 × 0.08 mm

#### Data collection

Bruker SMART CCD area-detector diffractometer
Absorption correction: multi-scan (*SADABS*; Bruker, 2000 ▶) *T*_min_ = 0.980, *T*_max_ = 0.992
7399 measured reflections
1412 independent reflections
1087 reflections with *I* > 2σ(*I*)
*R*_int_ = 0.043

#### Refinement

*R*[*F* ^2^ > 2σ(*F* ^2^)] = 0.064
*wR*(*F* ^2^) = 0.187
*S* = 1.07
1412 reflections
136 parameters
All H-atom parameters refinemed
Δρ_max_ = 0.29 e Å^−3^
Δρ_min_ = −0.30 e Å^−3^

### 

Data collection: *SMART* (Bruker, 2000 ▶); cell refinement: *SAINT-Plus* (Bruker, 2000 ▶); data reduction: *SAINT-Plus*; program(s) used to solve structure: *SHELXTL* (Sheldrick, 2008 ▶); program(s) used to refine structure: *SHELXL97* (Sheldrick, 2008 ▶); molecular graphics: *SHELXTL*; software used to prepare material for publication: *SHELXL97* and *PLATON* (Spek, 2009 ▶).

## Supplementary Material

Crystal structure: contains datablocks I, global. DOI: 10.1107/S1600536809031924/jh2093sup1.cif

Structure factors: contains datablocks I. DOI: 10.1107/S1600536809031924/jh2093Isup2.hkl

Additional supplementary materials:  crystallographic information; 3D view; checkCIF report

## Figures and Tables

**Table 1:** Hydrogen-bond geometry (Å, °)

*D*—H⋯*A*	*D*—H	H⋯*A*	*D*⋯*A*	*D*—H⋯*A*
C5—H3⋯O1^i^	0.96 (3)	2.48 (3)	3.396 (4)	159 (3)
C9—H7⋯O1^ii^	0.95 (3)	2.55 (4)	3.495 (4)	173 (3)

Symmetry codes: (i) 
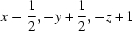
; (ii) 
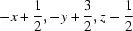
.

**Fig. 1.**
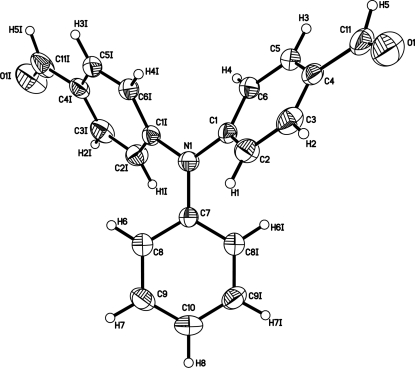
The molecular structure of (I), with atom labels and 30% probability displacement ellipsoids for non-H atoms. C1I to C6I,C8I,C9I,C11I,O1I and H1I to H7I were created by GROW and the symmetry code of "I" is -x, +y, 0.5 - z.

**Fig. 2.**
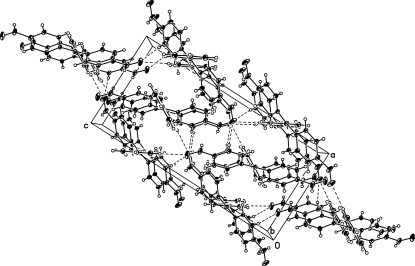
The packing of (I), viewed down the b axis.

## supplementary crystallographic information

### Comment

The popularity of tris(4-formylphenyl)amine as a building block is rapidly growing in materials chemistry (Thomas *et al.*, 2005). The title compound, (I) (Fig. 1), [C_20_H_15_NO_2_], was obtained unintentionally as the product of an attempted purification of tris(4-formylphenyl)amine.

The molecule of (I) has three phenyl rings, but the asymmetric unit contains only one half of (I). The ring (C7 to C10) makes a dihedral angle of 70.36 (8)° with ring (C1 to C6), and a dihedral angle of 70.22 (8)° with ring (C1^i^ to C6^i^) (symmetry code: (i) -*x*, +*y*, 0.5 - *z*). The dihedral angle of the latter two is 66.66 (8)°.

The *PLATON* program (Spek, 2009) suggests that there are no classic hydrogen bonds, but there are weak C—H···O hydrogen bonds (Table 2, Krishnamohan Sharma & Desiraju, 1994) between carbonyl oxygen and H atoms on the adjacent molecules, which link them into infinite three-dimensional network[Fig. 2].

### Experimental

Phosphorus oxychloride (POCl_3_) and *N*,*N*-dimethylformamide (DMF) were analytical reagent and used after the process of removing oxygen and water. Other organic solvents and common materials used for synthesis were used without further purification. The compound (I) was prepared by mixing 5.0 g triphenylamine and an ice-cooled mixture of POCl_3_(47.5 mL) and DMF(36.3 mL) under N_2_. The resulting mixture was stirred at 95°C for 4 h under N_2_. After cooling to room temperature,the mixture was poured into ice-water(1*L*),and basified with 1*M* NaOH. After filtration, the crude product was purified by column chromatography with petroleum ether/ethyl acetate (8/1,in volume ratio) to yield I(yellow transparent crystal). Elemental analysis Calcd: C 79.72, H 5.02, N 4.65%. Found: C 79.81, H 5.16, N 4.57%.

### Refinement

All the H atoms were located in the difference Fourier map and all parameters are refined independently.

### Crystal data

**Table:**

C_20_H_15_NO_2_	*D*_x_ = 1.253 Mg m^−^^3^
*M**_r_* = 301.33	Melting point = 417–419 K
Orthorhombic, *P**b**c**n*	Mo *K*α radiation, λ = 0.71073 Å
Hall symbol: -P 2n 2ab	Cell parameters from 378 reflections
*a* = 8.836 (2) Å	θ = 1.7–25.0°
*b* = 9.710 (2) Å	µ = 0.08 mm^−^^1^
*c* = 18.621 (4) Å	*T* = 298 K
*V* = 1597.6 (6) Å^3^	Block, yellow
*Z* = 4	0.32 × 0.18 × 0.08 mm
*F*(000) = 632	

### Data collection

**Table:**

Bruker SMART CCD area-detector diffractometer	1412 independent reflections
Radiation source: fine-focus sealed tube	1087 reflections with *I* > 2σ(*I*)
graphite	*R*_int_ = 0.043
π and ω scans	θ_max_ = 25.0°, θ_min_ = 2.2°
Absorption correction: multi-scan (*SADABS*; Bruker, 2000)	*h* = −10→10
*T*_min_ = 0.980, *T*_max_ = 0.992	*k* = −11→8
7399 measured reflections	*l* = −22→21

### Refinement

**Table:**

Refinement on *F*^2^	Primary atom site location: structure-invariant direct methods
Least-squares matrix: full	Secondary atom site location: difference Fourier map
*R*[*F*^2^ > 2σ(*F*^2^)] = 0.064	Hydrogen site location: difference Fourier map
*wR*(*F*^2^) = 0.187	All H-atom parameters refined
*S* = 1.07	*w* = 1/[σ^2^(*F*_o_^2^) + (0.0996*P*)^2^ + 0.4228*P*] where *P* = (*F*_o_^2^ + 2*F*_c_^2^)/3
1412 reflections	(Δ/σ)_max_ < 0.001
136 parameters	Δρ_max_ = 0.28 e Å^−^^3^
0 restraints	Δρ_min_ = −0.30 e Å^−^^3^

### Special details

**Table:**

Geometry. All e.s.d.'s (except the e.s.d. in the dihedral angle between two l.s. planes) are estimated using the full covariance matrix. The cell e.s.d.'s are taken into account individually in the estimation of e.s.d.'s in distances, angles and torsion angles; correlations between e.s.d.'s in cell parameters are only used when they are defined by crystal symmetry. An approximate (isotropic) treatment of cell e.s.d.'s is used for estimating e.s.d.'s involving l.s. planes.
Refinement. Refinement of *F*^2^ against ALL reflections. The weighted *R*-factor *wR* and goodness of fit *S* are based on *F*^2^, conventional *R*-factors *R* are based on *F*, with *F* set to zero for negative *F*^2^. The threshold expression of *F*^2^ > σ(*F*^2^) is used only for calculating *R*-factors(gt) *etc*. and is not relevant to the choice of reflections for refinement. *R*-factors based on *F*^2^ are statistically about twice as large as those based on *F*, and *R*- factors based on ALL data will be even larger.

### Fractional atomic coordinates and isotropic or equivalent isotropic displacement parameters (Å^2^)

**Table:**

	*x*	*y*	*z*	*U*_iso_*/*U*_eq_	
C1	0.0438 (3)	0.5517 (3)	0.31252 (11)	0.0446 (6)	
C2	0.1601 (3)	0.6013 (3)	0.35553 (15)	0.0587 (8)	
C3	0.1977 (4)	0.5311 (3)	0.41719 (16)	0.0670 (9)	
C4	0.1216 (3)	0.4133 (3)	0.43801 (13)	0.0593 (8)	
C5	0.0081 (3)	0.3648 (3)	0.39479 (14)	0.0573 (7)	
C6	−0.0305 (3)	0.4314 (3)	0.33246 (13)	0.0499 (7)	
C7	0.0000	0.7700 (3)	0.2500	0.0458 (8)	
C8	0.0651 (3)	0.8417 (3)	0.19405 (15)	0.0581 (8)	
C9	0.0634 (4)	0.9841 (3)	0.19400 (18)	0.0697 (9)	
C10	0.0000	1.0547 (5)	0.2500	0.0712 (12)	
C11	0.1559 (5)	0.3415 (4)	0.50518 (16)	0.0873 (12)	
N1	0.0000	0.6232 (3)	0.2500	0.0528 (8)	
O1	0.2531 (4)	0.3709 (3)	0.54591 (13)	0.1245 (12)	
H1	0.212 (3)	0.682 (3)	0.3407 (14)	0.067 (8)*	
H2	0.271 (4)	0.566 (4)	0.4436 (17)	0.089 (10)*	
H3	−0.041 (3)	0.282 (4)	0.4107 (16)	0.090 (10)*	
H4	−0.111 (3)	0.399 (3)	0.3032 (13)	0.059 (8)*	
H5	0.101 (4)	0.248 (5)	0.5133 (19)	0.113 (13)*	
H6	0.106 (3)	0.790 (3)	0.1563 (16)	0.071 (8)*	
H7	0.110 (3)	1.031 (3)	0.1554 (18)	0.088 (10)*	
H8	0.0000	1.153 (6)	0.2500	0.092 (15)*	

### Atomic displacement parameters (Å^2^)

**Table:**

	*U*^11^	*U*^22^	*U*^33^	*U*^12^	*U*^13^	*U*^23^
C1	0.0513 (14)	0.0464 (14)	0.0362 (12)	0.0041 (11)	−0.0018 (10)	−0.0007 (10)
C2	0.0626 (17)	0.0572 (17)	0.0563 (16)	−0.0078 (14)	−0.0105 (13)	−0.0025 (14)
C3	0.0714 (19)	0.073 (2)	0.0566 (17)	0.0136 (16)	−0.0257 (15)	−0.0160 (16)
C4	0.086 (2)	0.0492 (15)	0.0429 (14)	0.0234 (14)	−0.0042 (14)	−0.0046 (12)
C5	0.0769 (19)	0.0508 (16)	0.0441 (14)	0.0065 (14)	0.0073 (14)	0.0005 (12)
C6	0.0540 (15)	0.0510 (15)	0.0447 (13)	−0.0005 (12)	−0.0015 (12)	−0.0007 (12)
C7	0.0535 (19)	0.0444 (19)	0.0396 (17)	0.000	−0.0017 (15)	0.000
C8	0.0658 (17)	0.0573 (18)	0.0513 (15)	0.0000 (13)	0.0066 (13)	0.0003 (13)
C9	0.081 (2)	0.0585 (19)	0.0696 (19)	−0.0096 (15)	0.0021 (16)	0.0158 (16)
C10	0.080 (3)	0.044 (2)	0.090 (3)	0.000	−0.005 (2)	0.000
C11	0.138 (3)	0.077 (2)	0.0460 (17)	0.045 (2)	−0.017 (2)	−0.0119 (17)
N1	0.072 (2)	0.0447 (17)	0.0419 (15)	0.000	−0.0079 (14)	0.000
O1	0.181 (3)	0.117 (2)	0.0762 (16)	0.059 (2)	−0.0570 (19)	−0.0115 (15)

### Geometric parameters (Å, °)

**Table:**

C1—C2	1.389 (4)	C7—C8^i^	1.379 (3)
C1—C6	1.391 (4)	C7—C8	1.379 (3)
C1—N1	1.410 (3)	C7—N1	1.425 (4)
C2—C3	1.376 (4)	C8—C9	1.383 (4)
C2—H1	0.95 (3)	C8—H6	0.93 (3)
C3—C4	1.383 (4)	C9—C10	1.368 (4)
C3—H2	0.88 (3)	C9—H7	0.95 (3)
C4—C5	1.369 (4)	C10—C9^i^	1.368 (4)
C4—C11	1.464 (4)	C10—H8	0.95 (5)
C5—C6	1.372 (4)	C11—O1	1.181 (4)
C5—H3	0.96 (3)	C11—H5	1.04 (4)
C6—H4	0.95 (3)	N1—C1^i^	1.410 (3)
			
C2—C1—C6	119.1 (2)	C8^i^—C7—C8	119.4 (4)
C2—C1—N1	120.6 (2)	C8^i^—C7—N1	120.32 (18)
C6—C1—N1	120.3 (2)	C8—C7—N1	120.32 (18)
C3—C2—C1	119.2 (3)	C7—C8—C9	120.1 (3)
C3—C2—H1	122.3 (16)	C7—C8—H6	117.3 (17)
C1—C2—H1	118.5 (16)	C9—C8—H6	122.6 (17)
C2—C3—C4	121.7 (3)	C10—C9—C8	120.3 (3)
C2—C3—H2	117 (2)	C10—C9—H7	121 (2)
C4—C3—H2	121 (2)	C8—C9—H7	119 (2)
C5—C4—C3	118.4 (3)	C9^i^—C10—C9	119.9 (4)
C5—C4—C11	119.3 (3)	C9^i^—C10—H8	120.1 (2)
C3—C4—C11	122.2 (3)	C9—C10—H8	120.1 (2)
C4—C5—C6	121.1 (3)	O1—C11—C4	125.8 (4)
C4—C5—H3	115.9 (19)	O1—C11—H5	117 (2)
C6—C5—H3	122.9 (19)	C4—C11—H5	116 (2)
C5—C6—C1	120.3 (3)	C1—N1—C1^i^	121.0 (3)
C5—C6—H4	121.0 (16)	C1—N1—C7	119.50 (14)
C1—C6—H4	118.6 (16)	C1^i^—N1—C7	119.50 (14)

Symmetry codes: (i) −*x*, *y*, −*z*+1/2.

### Hydrogen-bond geometry (Å, °)

**Table:**

*D*—H···*A*	*D*—H	H···*A*	*D*···*A*	*D*—H···*A*
C5—H3···O1^ii^	0.96 (3)	2.48 (3)	3.396 (4)	159 (3)
C9—H7···O1^iii^	0.95 (3)	2.55 (4)	3.495 (4)	173 (3)

Symmetry codes: (ii) *x*−1/2, −*y*+1/2, −*z*+1; (iii) −*x*+1/2, −*y*+3/2, *z*−1/2.
